# Elucidation of the tumoritropic principle of hypericin

**DOI:** 10.1038/sj.bjc.6602512

**Published:** 2005-04-20

**Authors:** M Van de Putte, T Roskams, J R Vandenheede, P Agostinis, P A M de Witte

**Affiliations:** 1 Laboratorium voor Farmaceutische Biologie, Faculteit Farmaceutische Wetenschappen, KU Leuven, Van Evenstraat 4, B-3000 Leuven, Belgium; 2 Afdeling Histochemie en Cytochemie, Faculteit Geneeskunde, KU Leuven, Belgium; 3 Afdeling Biochemie, Faculteit Geneeskunde, KU Leuven, Belgium

**Keywords:** photodynamic therapy, hypericin, tumoritropic behaviour, perfusion, permeability, lipoproteins

## Abstract

Hypericin is a potent agent in the photodynamic therapy of cancers. To better understand its tumoritropic behaviour, we evaluated the major determinants of the accumulation and dispersion of hypericin in subcutaneously growing mouse tumours. A rapid exponential decay in tumour accumulation of hypericin as a function of tumour weight was observed for each of the six tumour models investigated, and a similar relationship was found between tumour blood flow and tumour weight. Moreover, there was a close correlation between the higher hypericin uptake in RIF-1 tumours compared to R1 tumours and tumour vessel permeability. To define the role of lipoproteins in the transport of hypericin through the interstitial space, we performed a visual and quantitative analysis of the colocalisation of hypericin and DiOC_18_-labelled lipoproteins in microscopic fluorescent overlay images. A coupled dynamic behaviour was found early after injection (normalised fluorescence intensity differences were on the whole less than 10%), while a shifted pattern in localisation of hypericin and DiOC_18_ was seen after 24 h, suggesting that during its migration through the tumour mass, hypericin is released from the lipoprotein complex. In conclusion, we were able to show that the tumour accumulation of hypericin is critically determined by a combination of biological (blood flow, vessel permeability) and physicochemical elements (affinity for interstitial constituents).

Photodynamic therapy (PDT) involves the local or systemic administration of a photosensitising drug that, upon light irradiation and in the presence of oxygen, results in tumour destruction (Dolmans *et al*, 2003). We have recently been focusing on hypericin, a natural compound isolated from Hypericum plants (Lavie *et al*, 1995a), as a potent photosensitiser with a high antitumoral PDT efficacy (Chen and de Witte, 2000; Chen *et al*, 2001, 2002). In the course of our study, we found that the compound accumulated to a large extent in tumour tissues. For instance, after systemic administration (i.p. 5 mg kg^−1^ hypericin), a 16-fold higher concentration of hypericin in tumour tissue *vs* surrounding healthy tissue (skin, muscle) was found in a subcutaneous P388 lymphoma tumour model growing in DBA/2 mice (Chen and de Witte, 2000). A fast clearance of hypericin in the liver, spleen, kidney and plasma was observed within 6 h, while the peak concentration of hypericin in the tumour (maximal 8.7% of the injected dose per gram tissue (% ID g^−1^)) occurred at 24–48 h after drug administration. To confirm these data, a study using C3H mice bearing subcutaneous RIF-1 fibrosarcoma tumours was performed. The tumour drug concentration increased rapidly over the initial hours and peaked (5.5% ID g^−1^) approximately 6 h after i.v. administration (Chen *et al*, 2001).

The tumoritropic characteristics of hypericin, therefore, imply that some (radio)labelled derivatives of the compound could be applied in the field of clinical radiodiagnosis, radiotherapy (^131^I-labelled) and possibly also in magnetic resonance imaging (MRI). In order to better understand the mechanistic background, the present paper addresses basic aspects and principles of the accumulation of hypericin in malignant tissue. Previous results have shown that, compared to normal cells, isolated malignant cells intrinsically do not take up more hypericin (Kamuhabwa *et al*, 2000). Therefore, the tumoritropic behaviour of hypericin should be envisioned as the result of molecular interactions with specific *in vivo* environmental, vascular and tumour tissue properties. In this paper, the tumour tissue accumulation of hypericin was examined as a function of tumour weight and a correlation with tumour perfusion and tumour vessel permeability was explored. Furthermore, we also investigated the intratumoral distribution of hypericin and its association with lipoproteins, aimed at defining the role of lipoproteins as regards the transport through the interstitial space.

## MATERIALS AND METHODS

### Animals and tumour system

The following tumour models were used: (a) mouse RIF-1 (radiation-induced fibrosarcoma) cells (kindly provided by Dr F Stewart, The Netherlands Cancer Institute, The Netherlands) subcutaneously (s.c.) grafted in female C3H/Km mice, (b) mouse MH22A hepatoma cells (kindly provided by Dr Z Luksiene, Institute of Materials Science and Applied Research, Lithuania) s.c. grafted in female athymic nude mice, (c) human CaCo-2 colon carcinoma cells (kindly provided by Dr P Augustijns (KU Leuven)) s.c. grafted in female athymic nude mice, (d) human A431 cervix carcinoma cells (obtained from American Type Culture Collection (ATCC)) s.c. grafted in female athymic nude mice, (e) AY27 TCC (transitional cell carcinoma) rat cells (originally developed by Drs S Selman and JA Hampton (Ohio Medical College)) s.c. grafted in female athymic nude mice and (f) R1 rhabdomyosarcoma rat cells (kindly provided by Dr W Landuyt (KU Leuven)) s.c. grafted in female athymic nude mice.

Tumour cells (2 × 10^6^) were inoculated s.c. on the depilated lower dorsum of female mice (weight range 21–25 g, purchased from Charles River Laboratories (France) or B&K Grimston (England)). Tumours were grown to different surface diameters ranging from 2 to 9 mm and to thicknesses ranging between 2 and 5 mm, as measured by a calliper. These dimensions covered tumour weights from ca 5 to 200 mg.

All aspects of the animal experiment and husbandry were carried out in compliance with national and European regulations and were approved by the Animal Care and Use Committee of KU Leuven.

Statistical analysis was performed using Prism 4.00, GraphPad Software, San Diego, USA.

### Hypericin accumulation in tumour tissue

Tumour-bearing animals were killed 6 h after i.v. tail injection of hypericin (5 mg kg^−1^). Hypericin (synthesised from emodin anthraquinone according to Falk *et al* (1993)) was dissolved in a mixture of 25% dimethylsulphoxide (DMSO), 25% polyethylene glycol (PEG) 400 and water (2 mg ml^−1^) immediately before injection. Tumour tissues were harvested, weighed and frozen at −20°C until determination of the hypericin content. Similar tissue samples were taken from control mice. Extraction and quantification of tissue hypericin concentrations were performed as previously described (Chen *et al*, 2001).

### Tumour blood perfusion

The RIF-1 and R1 tumour-bearing animals were used to quantify the tumour perfusion by means of spectrofluorometric determination of tumour FITC-dextran uptake (fluorescein isothiocyanate dextran, *M*_r_ 2 × 10^6^, obtained from Sigma, St Louis, MO, USA), as described (Chen *et al*, 2002).

### Tumour vessel permeability

The RIF-1 and R1 tumour-bearing animals were used to assess the tumour vessel permeability by a modification of the procedure described by Graff *et al* (2001). Evans blue dye (Sigma, St Louis, MO, USA) was dissolved in PBS (5 mg ml^−1^) and injected i.p. in tumour-bearing mice (25 mg kg^−1^). After 48 h, the animals were killed and the dissected and weighed tumour tissues dissolved in 1 ml of tissue solubiliser (Soluene 350; Packard Industries, Downers Grove, IL, USA) at 37°C overnight. The solution was allowed to cool before the addition of 2 ml of ethyl acetate (Fisher Scientific, UK) and 2 ml of 1 N HCl. Absorption of the upper phase was read at 626 nm using a UV/Visible Spectrophotometer (Ultrospec 2000 Pharmacia Biotech, Amersham Biosciences, Uppsala Sweden). Concentrations were determined from a standard curve of Evans blue dye.

### Intratumoral localisation of hypericin and DiOC_18_

The intratumoral localisation of hypericin and DiOC_18_ (3,3′-dioctadecyloxacarbocyanine, Molecular Probes Inc., Eugene, OR, USA) was investigated by i.v. administering hypericin (5 mg kg^−1^), immediately followed by DiOC_18_ (5 mg kg^−1^) to RIF-1 tumour-bearing animals (tumour weight range 150–200 mg) 5 min, 2 h, 6 h or 24 h before being killed. Prior to injection, DiOC_18_ was suspended (2 mg ml^−1^) in 40% propylene glycol, 10% ethanol, 4% Tween 80 and water, after which the mixture was sonicated at 60°C for 2 h. In another set of experiments, FITC-dextran (300 mg kg^−1^, in PBS 30 mg ml^−1^) was administered 2 min before killing the RIF-1 tumour-bearing animals that had received hypericin (5 mg kg^−1^).

Tumour samples were immediately mounted in medium (Tissue Tek embedding medium, Miles Inc., Elkhart, IN 46515, USA) and immersed in liquid nitrogen. Different serial cryostat sections (5 *μ*M slices) were taken from each tumour. The first of two serial sections was stained with hematoxylin and eosin (H&E) and the second was examined by fluorescence microscopy (Axioskop 2 plus equipped with a light-sensitive charge-coupled device digital camera (Carl Zeiss, Göttingen, Germany)). To specifically visualise hypericin, the Zeiss filter set 14 (ex: BP 510–560 nm, em: LP 590 nm) was used, whereas the distribution of DiOC_18_ or FITC-dextran was examined with Zeiss filter set 10 (ex: BP 450–490 nm, em: BP 515–565 nm).

Overlay fluorescence images were quantitatively analysed using a KS imaging software system (Carl Zeiss, Göttingen, Germany) by subdividing the images in 2269 square fields of 38.7 *μ*M^2^ (24 pixels/field) and by measuring the average fluorescence intensity per field for hypericin and DiOC_18_, respectively. The data were normalised for the maximal fluorescence intensity of each compound, and expressed as percentage fluorescence intensity (% f.i.). From these field-by-field data, scattergrams were constructed with axes representing the % f.i. of each compound. In addition, the absolute field-by-field differences in fluorescence intensity for hypericin and DiOC_18_ (i.e. Δ=∣(% f.i._hypericin_–% f.i._DiOC18_)∣) were calculated. The differences were grouped in fractions with increments of 10% and the percentage of fields corresponding to each of the fractions was determined. In total, 15 overlay fluorescence images randomly taken throughout different tumours (*n*=3) were analysed for each time interval.

## RESULTS

### Tumour accumulation of hypericin

**Figure 1:**
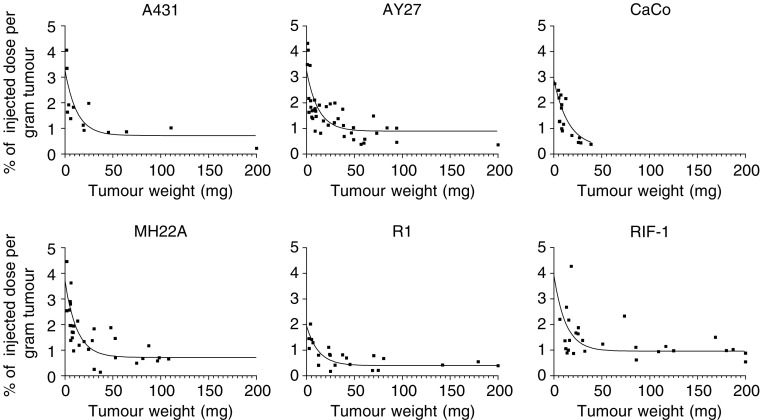
Accumulation of hypericin, expressed as % of injected dose per gram of tumour, as a function of tumour weight. Animals bearing A431, AY27, CaCo-2, MH22A, R1 or RIF-1 tumours were killed at 6 h after i.v. injection (tail) of hypericin (5 mg kg^−1^). Data points were fitted using one-phase exponential decay.

**Figure 2:**
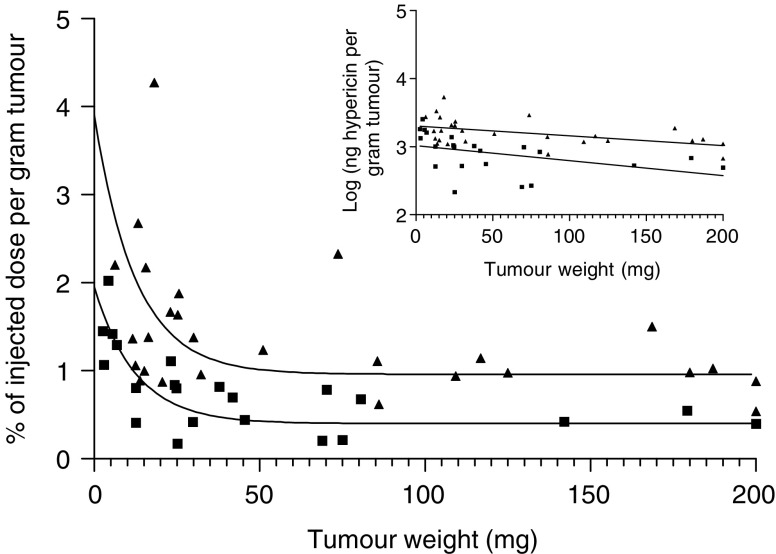
Comparison of the relative accumulation of hypericin in R1 (▪) and RIF-1 tumours (▴). The accumulation of hypericin, expressed as % of injected dose per gram of tumour is shown as a function of tumour weight. Data points were fitted using one-phase exponential decay. The logarithm of the amount of hypericin recovered per gram of tumour tissue as a function of tumour weight is depicted in the inset (for RIF-1: *y*=−0.001436*x*+3.305; for R1: *y*=−0.002194*x*+3.015). The data were statistically compared after linearisation using the two-way ANCOVA test (analysis of covariance). The difference in relative accumulation of hypericin in R1 and RIF-1 tumours was extremely significant (*P*-value< 0.0001).

After systemic administration of hypericin, its uptake in tumour tissue was studied as a function of tumour weight in six tumour models. As previous results using the RIF-1 tumour model had demonstrated, a peak concentration of hypericin in tumour tissue between 4 and 8 h after intravenous injection, a 6 h interval between administration and analysis was used in all cases. From Figure 1 it can be seen that, hinging on the tumour size, large intratumoral differences in hypericin accumulation exist. Typically, small tumours tended to accumulate three to four times more of hypericin relative to their weight, as compared to larger tumours (ranging from 50 to 200 mg). As a matter of fact, for each tumour model investigated, a rapid exponential decay in hypericin accumulation was observed from the smallest tumours followed by a plateau phase, on the average starting from a tumour weight of 50 mg. Of interest, when comparing intertumoral dissimilarities, a difference in overall tumour accumulation was found between the mouse RIF-1 fibrosarcoma and rat R1 rhabdomyosarcoma tumours (see Figure 2).

### Tumour blood perfusion and vessel permeability

**Figure 3:**
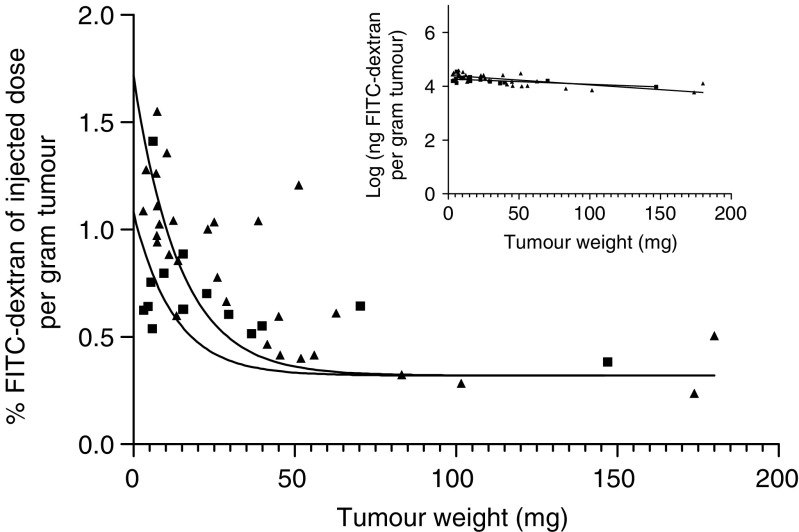
Perfusion of RIF-1 (▴) and R1 (▪) tumours. The amount FITC-dextran extracted is expressed as % of injected dose per gram of tumour, as a function of tumour weight. FITC-dextran (100 mg kg^−1^) was i.v. injected 2 min before killing. Data points were fitted using one-phase exponential decay. The logarithm of the amount of FITC-dextran recovered per gram tumour tissue as a function of tumour weight is depicted in the inset (for RIF-1: *y*=−0.003575*x*+4.408; for R1: *y*=−0.001982*x*+4.273). The data were statistically compared after linearisation using the two-way ANCOVA test (analysis of covariance). No significant difference between the perfusion of RIF-1 and R1 tumours was observed (*P*-value: 0.064).

To further evaluate the intratumoral and intertumoral differences in hypericin accumulation disclosed in R1 and RIF-1 tumours, we analysed and compared their relative perfusion as well as their vessel permeability as a function of tumour weight. To measure tumour perfusion, FITC-dextran was i.v. injected 2 min before the animal was killed . At this short interval, extravasation of the FITC-dextran complex is negligible (Shockley *et al*, 1992) and therefore the fluorescent tracer is entirely confined to the lumen of the (tumour) blood vessels. The amount of dye extracted from the tumour was expressed as % of the injected dose per gram of tumour as a function of tumour weight (see Figure 3). Intratumoral differences similar to the one found in case of the hypericin accumulation can be seen for both tumour types, that is, a weight-dependent exponential decay in tumour blood flow followed by a plateau phase from tumour weights of 50 mg on. As there was no significant difference between RIF-1 and R1 tumours (see Figure 3), an intertumoral variability in perfusion could not be found.

**Figure 4:**
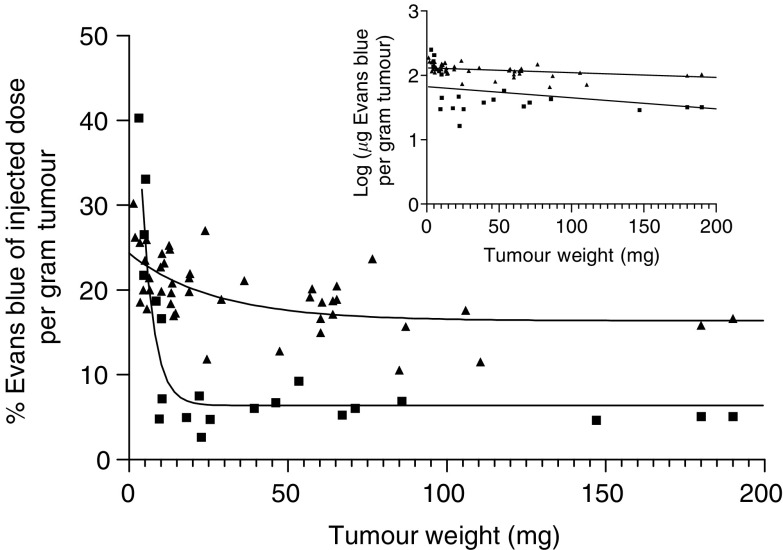
Extravasation of Evans blue in RIF-1 tumours (▴) and R1 tumours (▪), expressed as % of injected dose per gram of tumour, as a function of tumour weight. Data points were fitted using one-phase exponential decay. The logarithm of the amount of Evans blue recovered per gram tumour tissue as a function of tumour weight is depicted in the inset (for RIF-1: *y*=−0.0007355*x*+2.116; for R1: *y*=−0.001724*x*+1.826). The data were statistically compared after linearisation using the two-way ANCOVA test (analysis of covariance). The difference in vessel permeability in R1 and RIF-1 tumours was extremely significant (*P*-value< 0.0001).

Tumour vessel permeability was measured by i.p. injection of Evans blue dye, a dye with a strong affinity for serum albumin. Extravasation of this dye is based on vessel permeability, which makes it a good marker for permeability measurements (Graff *et al*, 2001). Figure 4 shows the % of injected dose per gram of tumour *vs* tumour weight, 48 h after administration. Except for small tumours (<20 mg), the relative amount of Evans blue recovered from one tumour type was similar over a large range of tumour weight. However, major intertumoral differences between the permeability of vessels present in RIF-1 and R1 tumours were found.

### Intratumoral localisation of hypericin

To track the intratumoral fate of hypericin, a fluorescence microscopy study was performed on sections of tumour biopsies taken at different time points after systemic administration of the compound to RIF-1 tumour-bearing animals. Since blood-borne hypericin mainly associates with high-density lipoproteins (HDL) and other lipoproteins (Chen *et al*, 2001), it was of interest to verify whether the compound colocated intratumorally with the lipoproteins upon intravenous administration. For that purpose, DiOC_18_ was simultaneously injected with hypericin into the bloodstream. DiOC_18_ is a green fluorescent analogue of DiIC_18_ (Pitas *et al*, 1981), a marker that by means of its very lipophilic C_18_ moieties avidly binds to lipoproteins without altering their affinity for the receptors.

**Figure 5:**
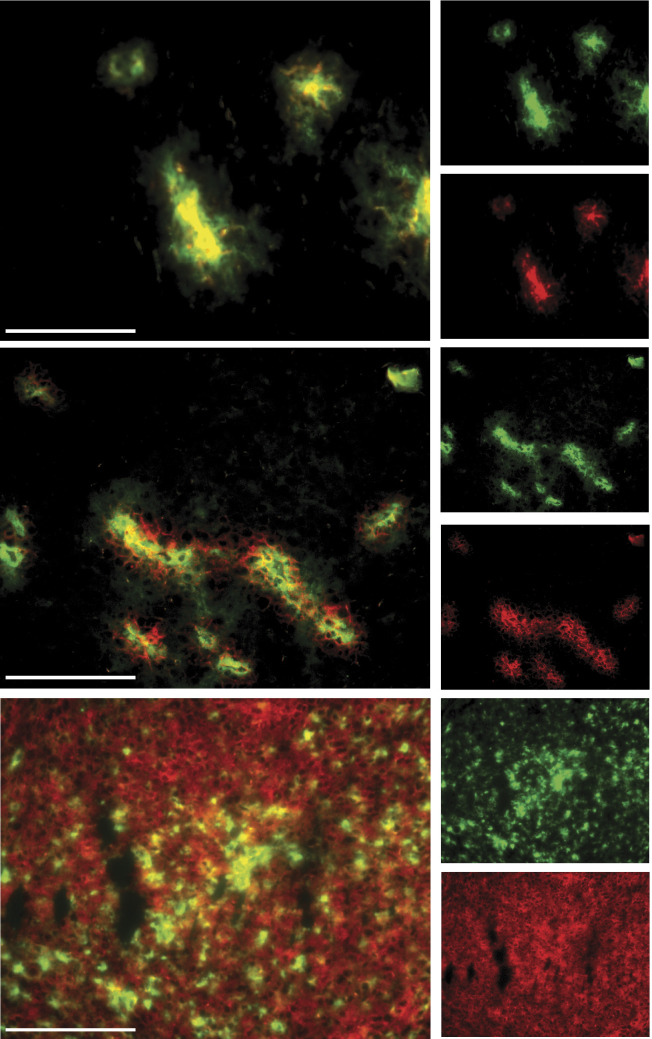
Fluorescence photomicrographs of 5 *μ*m RIF-1 mouse tumour sections sampled at 5 min (**A**), 2 h (**B**) and 24 h (**C**) after i.v. injection of 5 mg kg^−1^ hypericin (red fluorescence) and 5 mg kg^−1^ DiOC_18_-labelled lipoproteins (green fluorescence). Overlay pictures show the combination of individual photomicrographs of hypericin in the left column, and of DiOC_18_ in the right column. Scale bar=100 *μ*M.

Fluorescence microscopic analysis of tumour tissue, taken 5 min, 2, 6 and 24 h after administration of the compounds, revealed a shifting pattern, as a function of time, in the localisation of labelled lipoproteins and hypericin. At 5 min, DiOC_18_ and hypericin were still confined to the luminal space of tumoral blood vessels (Figure 5A), as shown in separate experiments with FITC-dextran that was injected 2 min before killing the animals (results not shown). Figure 5B shows the situation after 2 h, where red (hypericin) and green (DiOC_18_) fluorescence are apparent in the vessels and in the perivascular region. Conversely, at 24 h, a more homogeneous distribution is observed for hypericin in contrast to DiOC_18_-labelled lipoproteins that show an irregular spreading (Figure 5C).

**Figure 6:**
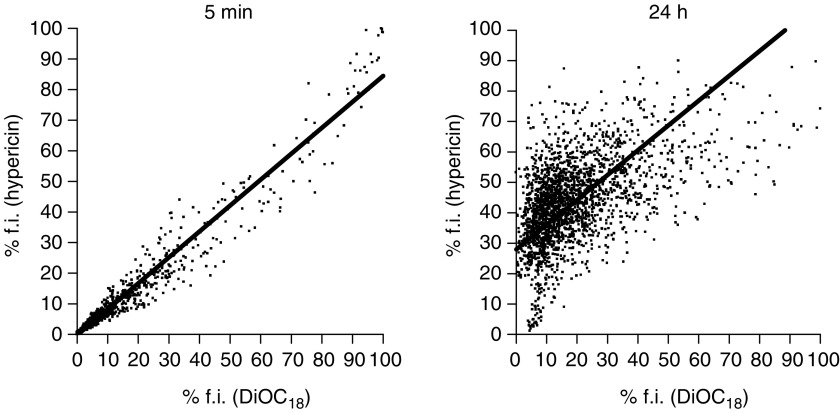
Scattergrams representing 2269 field-by-field relationships between normalised fluorescence intensities (% f.i.) of hypericin and DiOC_18_ at 5 min (*r*=0.99) and 24 h (*r*=0.52) after coadministration of the compounds. Results of representative fluorescent overlay images are shown.

**Figure 7:**
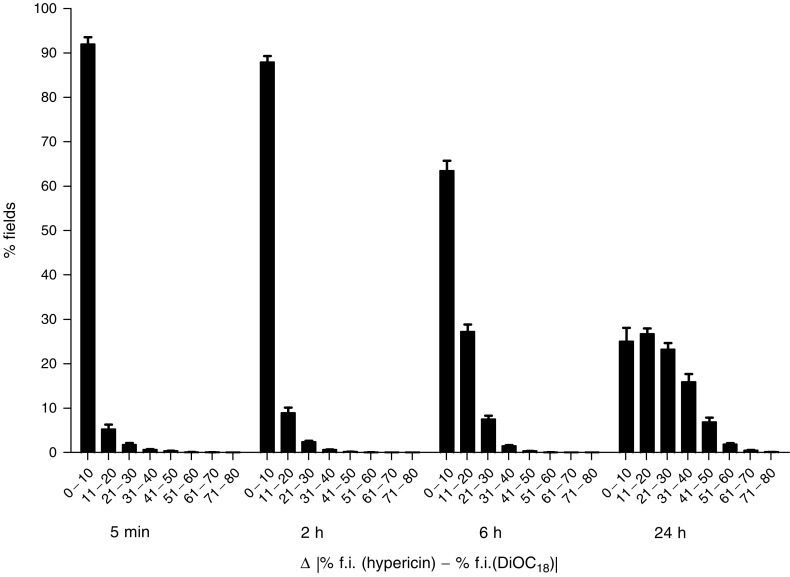
Time-dependent distribution of percentages of fields as a function of absolute differences (Δ) between normalised fluorescence intensities (% f.i.) of hypericin and DiOC_18_. Δ Values were grouped in fractions with increments of 10%. Data are expressed as mean±s.d. (average of the analysis of three tumours and 15 fluorescent overlay images per tumour). Relative field counts for Δ values between 80 and 100% were zero at all time points. Statistical analyses of the differences are shown in Table 1.

Quantitative measurements of colocalisation of hypericin and DiOC_18_ were examined by a field-by-field analysis of fluorescent overlay images. Scattergrams revealed a good correlation between the percentage fluorescence intensity of hypericin and DiOC_18_-labelled lipoproteins at short intervals after coadministration of the compounds, whereas a poor correlation was observed at the later time points (Figure 6). For each time point, the percentage of fields that fall within fractions of grouped absolute differences (Δ) was scored (Figure 7, Table 1). A time-dependent shift in the distribution among the fractions can be seen, with an increased amount of larger differences between both compounds at longer time intervals. For instance, at 5 min and 2 h, 92±6.1 and 88±5.6% (mean±s.d.), respectively, of the fields had Δ values less than 10%, indicating that at these time points the distribution of fluorescence between hypericin and DiOC_18_ was similar. However, 6 h after injection, significantly more fields displayed higher Δ values and at 24 h the majority of the fields exhibited Δ values of more than 10%.

## DISCUSSION

A basic understanding of the tumoritropic behaviour of hypericin would not only support the construction of hypericin derivatives with optimised PDT characteristics, but also sustain the development of labelled derivatives to extend the application of hypericins beyond the field of PDT. We therefore set out to gain a better insight in the major determinants of the accumulation and dispersion of hypericin in tumours.

Our study points out that, at least in the 5–50 mg tumour weight range, the extent of hypericin uptake depends on the tumour weight, while in larger tumours the uptake remains constant. In the two models investigated (R1, RIF-1), an identical relationship was found between tumour blood flow and tumour weight. The fact that small tumours are relatively better perfused than larger ones has been documented before (Tozer *et al*, 1990; Hering *et al*, 1995). Since both the hypericin accumulation and the blood flow hinge to the same extent on the tumour weight, our data suggest that the hypericin accumulation in tumour tissue is critically dependent on the extent of local blood perfusion. Consistent with this hypothesis, our fluorescence microscopy analysis of RIF-1 tumour sections revealed a more homogeneous vessel distribution in small tumours, with a high percentage of functional vessels and a lack of necrotic areas. In contrast, large tumours have a more heterogeneous vessel distribution, with well-perfused regions in the periphery and less blood flow in the centrally located viable tumour regions. As a consequence, hypericin mainly accumulates in the periphery of these tumours (results not shown). Our results therefore support the concept that, especially in larger tumours, the intratumoral blood flow distribution is rather heterogeneous, both spatially and temporally (Jain, 2001).

Furthermore, we consistently found that the hypericin uptake in RIF-1 tumours was about twice as high as in R1 tumours. Since this difference cannot be accounted for by a dissimilar tumour perfusion, we investigated the permeability of the tumour vessels involved.

Extravasation of plasma constituents by means of convective currents across the microvascular wall originates from the incomplete or totally missing endothelial lining of the rapidly formed vessels in tumours growing beyond a mass of 10^6^ cells (Feng *et al*, 2000; Hashizume *et al*, 2000; Kuszyk *et al*, 2001). Since Evans blue accumulated more in RIF-1 than in R1 tumours over a large tumour weight range, it could be concluded that the larger amount of marker extravasated in RIF-1 tumours was due to an increased permeability of the tumour vessels. Hence, it is likely that the relatively low leakiness of the R1 tumour vessels explains the lower uptake of hypericin in R1 as compared to RIF-1 tumours. A similar correlation between tumoral photosensitiser uptake and tumour vessel permeability has been reported before (Roberts and Hasan, 1993).

Importantly, it was reported that hypericin binds to human lipoproteins at high molar ratios (up to 467 and 14 moles per 10^4^ Da of low-density lipoproteins (LDL) and high-density lipoproteins (HDL), respectively) (Lavie *et al*, 1995b). In contrast to human plasma, mouse plasma contains a high HDL/LDL ratio (Kessel and Woodburn, 1993), so that mainly the hypericin–HDL complex is formed in the bloodstream of mice (results not shown). Once extravasated, the lipoproteins tagged with numerous hypericin molecules become embedded in the tumour interstitial fluid. At this stage, the hypericin–lipoprotein complexes can follow two routes leading to hypericin uptake and accumulation in tumour cells. Firstly, hypericin can comigrate with the lipoprotein microparticles throughout the interstitial space, followed by receptor-mediated intracellular uptake. Of importance here, a correlation exists between the extent of association of some classes of photosensitisers with LDL and their efficiency of tumour targeting (Jori and Reddi, 1993). Alternatively, after collision of the microparticles with interstitial proteins or tumour cell membranes, hypericin can be released from the lipoprotein complex, followed by a lipoprotein-independent diffusion and intracellular uptake of the compound. A similar *in vitro* cellular uptake of hypericin in serum-free conditions has been documented (Siboni *et al*, 2002).

To investigate which route prevails, lipoproteins were marked with DiOC_18_, a compound that virtually irreversibly associates with lipoproteins. After simultaneous administration of hypericin and DiOC_18_ to mice bearing RIF-1 tumours, fluomicroscopic analysis of tumour sections revealed that both compounds initially behaved similarly, indicating that hypericin and lipoproteins comigrate. However, later time intervals showed obvious differences in their intratumoral localisation. Thus, while the lipoproteins seem to be limited in their capacity to migrate through the interstitial space, hypericin spreads rather homogeneously over vascularised areas.

These results suggest that during its migration through the tumour mass, hypericin is released from the lipoprotein complex. In support of this notion, it is worth mentioning that unlike the lipophilic DiOC_18_, hypericin is an amphiphilic compound that preferentially locates to the polar aprotic zone adjacent to the lipid–water interface (Lenci *et al*, 1995; Weitman *et al*, 2001). The phospholipid moiety in lipoproteins is situated at the surface of the microparticle, thereby allowing the associated hypericin molecules to dynamically interact with the immediate surrounding. This surrounding consists of phospholipid bilayers of plasma membranes of cancer cells and immune cells, and of proteins like collagen which are abundantly present in the interstitial space (Jain, 1987). Interestingly, it has been shown that hypericin associates with collagen resulting in a photodynamic effect, while another photosensitiser Chlorin e_6_ was ineffective (Yova *et al*, 2001).

In conclusion, the results of the present study indicate that the tumour accumulation of hypericin is critically determined by a combination of tumour-dependent and compound-dependent factors. As far as the tumour tissue is concerned, it is clear that an adequate vascularisation, blood flow and vessel permeability, all contribute to the ability of hypericin to accumulate in the tumour mass. On the other hand, once extravasated and locally delivered by a lipoprotein carrier, the compound itself can dramatically affect the level of its migration throughout the interstitial zone. Our study reveals that hypericin may have an affinity for some constituents typically present in the interstitium, and that there probably is a partitioning of the compound over lipoproteins, structural proteins and lipid bilayers of cancer and immune cells. This partitioning allows hypericin to spread rather homogeneously over the tumour mass.

The present study shows that hypericin accumulates less in tumoral sites with a poorly developed or afunctional vascular system, typical of larger tumours. Of importance, since the plasma clearance rate of hypericin is fast, following a two-phase exponential decay with half-lives of only 0.08 and 1.4 h (Chen *et al*, 2001), prolonging the exposure time of tumour tissue to blood-borne hypericin, that is by repeated i.v. injections or by intraperitoneal hypericin pumps, could result in an increased tumoral uptake and an improved homogeneous diffusion over the whole tumour mass. This is an interesting possibility, which is currently under investigation in our laboratory.

## Figures and Tables

**Table 1:** Statistical comparison of means of percentages of fields

**Δ (%)**	**5 min**	**2 h**	**6 h**	**24 h**
0–10	2 h^*^ 6 h^***^ 24 h^***^	^*^5 min 6 h^***^ 24 h^***^	5 min^***^ 2 h^***^ 24 h^***^	5 min^***^ 2 h^***^ 6 h^***^
11–20	6 h^***^ 24 h^***^	6 h^***^ 24 h^***^	5 min^***^ 2 h^***^	5 min^***^ 2 h^***^
21–30	6 h^***^ 24 h^***^	6 h^**^ 24 h^***^	5 min^***^ 2 h^**^ 24 h^***^	5 min^***^ 2 h^***^ 6 h^***^
31–40	24 h^***^	24 h^***^	24 h^***^	5 min^***^ 2 h^***^ 6 h^***^
41–50	24 h^***^	24 h^***^	24 h^***^	5 min^***^ 2 h^***^ 6 h^***^
51–60	NS	NS	NS	NS
61–70	NS	NS	NS	NS
71–80	NS	NS	NS	NS

Levels of significance are indicated as: ^*^P<0.05, ^**^P<0.01, ^***^P<0.001. For instance, ^***^6 h (as mentioned in the 5 min column) means that the mean of % fields in the specific Δ fraction at 5 min is statistically different from the mean of % fields in the same Δ fraction calculated at 6 h, with *P*<0.001. NS=not significant. Statistical analysis was performed using the two-way ANOVA with Bonferroni test.
